# Comparative metabolomic analysis of leaves and kernels in wild type and *Zmsps2* mutant

**DOI:** 10.1016/j.fochms.2025.100343

**Published:** 2025-12-11

**Authors:** Jianting Lin, Yanchao Du, Haoxuan Jiang, Huating Zhao, Bo Wang, Faqiang Feng

**Affiliations:** a Guangdong Provincial Key Laboratory of Plant Molecular Breeding*,* South China Agricultural University*,* Guangzhou 510642*,* China; b Sweet and Waxy Corn Research Lab, Sorghum Research Institute*,* Shanxi Agricultural University*,* Jinzhong 030600*,* China

**Keywords:** ZmSPS2, Galactose metabolism, Metabolomic analysis, Sweet corn

## Abstract

The solanesyl diphosphate synthase gene *ZmSPS2* is known to modulate terpenoid metabolism and tocopherol biosynthesis in maize (*Zea mays* L.), though its system-wide metabolic effects remain poorly understood. In this study, a widely targeted metabolomic analysis revealed significant impacts of the *Zmsps2* mutation on metabolic networks in both leaves and kernels at 20 days after pollination (DAP). A total of 2531 metabolites were detected, primarily comprising lipids, organoheterocyclic compounds, and benzenoids. Differential metabolite analysis identified 453 significantly altered metabolites in leaves and 334 in kernels. In leaves, differential metabolites were enriched in four metabolic pathways including zma00051 (Fructose and mannose metabolism), zma00520 (Amino sugar and nucleotide sugar metabolism), zma00941 (Flavonoid biosynthesis), and zma00052 (Galactose metabolism). Four significant pathways were enriched in kernels, including zma02010 (ABC transporters), zma00052 (Galactose metabolism), zma00591 (Linoleic acid metabolism), and zma01230 (Biosynthesis of amino acids). This study demonstrates that the *Zmsps2* mutation triggers tissue-specific metabolic alterations: enhancing monosaccharide-driven energy supply in leaves, while promoting accumulation of protective sugars in kernels. These findings provide new insights into the regulation of metabolic profile by the *Zmsps2* mutation.

## Highlights

• *Zmsps2* mutation impacts maize metabolic networks, with 453/334 differential metabolites in leaf/kernel.
• *Zmsps2* mutation enhances monosaccharide energy supply in leaves, and increases protective sugars in kernels.
• Galactose metabolism is co-enriched in *Zmsps2* mutant leaves/kernels with distinct metabolic directions.

## 1 Introduction

As a globally vital cereal crop, maize (*Zea mays* L.) has experienced continuous expansion in cultivation to meet escalating food demands from population growth. With the global population exceeding 8 billion, there is increasing emphasis on improving both yield and nutritional quality, particularly regarding the development of α-tocopherol-enriched varieties. This enhancement has become particularly crucial for addressing vitamin E deficiency affecting approximately 1 billion people worldwide (Maman et al., 2023; Zhang et al., 2020). Maize kernels naturally contain various tocopherol isomers including γ- and δ-tocopherols, yet exhibit relatively low levels of α-tocopherol, the most biologically active form (Gunjevic et al., 2023). As lipid-soluble antioxidants, tocopherols demonstrate significant therapeutic potential in modulating childhood asthma and immune function (Xiong et al., 2023). Notably, epidemiological studies reveal strong correlations between serum α-tocopherol levels and metabolic health markers such as uric acid regulation in Asian populations (Kim, Choi, Kang, Kim, & Lee, 2020). Genetic improvement of α-tocopherol content through selective breeding represents a strategic approach to enhance maize's nutritional profile. Such advancements hold particular significance for animal feed formulations and human dietary interventions, where optimized vitamin E content could substantially mitigate global micronutrient deficiencies (Hossain et al., 2023; Zhang et al., 2020).

Despite decades of research on maize tocopherols, their biosynthetic regulatory networks remain incompletely characterized. Current evidence indicates that tocopherol synthesis requires coordinated expression of multiple genes (*VTE4*, *crtRB1*) and direct regulation by key enzymatic activities such as *HPPD* (Liu et al., 2024; Xu et al., 2019a). Overexpression of *ZmTMT* (γ-tocopherol methyltransferase) has demonstrated critical roles in enhancing α-tocopherol accumulation in transgenic *Arabidopsis* and maize seeds (Zhang et al., 2020). However, the regulatory mechanisms underlying chloroplast precursor supply pathways—such as the phytol cycle—and methyltransferase activity have not been fully elucidated (Herr et al., 2025; Zhan et al., 2019). Addressing these key scientific questions will provide a theoretical foundation for the biofortification breeding of maize.

In plants, solanesyl diphosphate synthase (SPS) serves as the pivotal enzyme for synthesizing C45 isoprenoid chains, generating precursor molecules for plastoquinone (PQ-9) and ubiquinone biosynthesis (Yan et al., 2017). The *SPS* gene family exhibits remarkable conservation across plant species, with encoded proteins typically containing dual aspartate-rich domains (DDxxD) that display significant homology to SPS enzymes in Solanaceae and other taxa (Yan et al., 2016). Studies in *Arabidopsis* demonstrate that Overexpression of *SPS1* elevates plastoquinone content and enhances photoxidative stress tolerance through redox state modulation of the plastoquinone pool (Ferretti et al., 2018; Ksas et al., 2018). In contrast, rice *OsSPS2* interacts with flavin-binding protein *OsFBN5* to mediate PQ-9 solanesyl chain elongation, a mechanism conserved in monocots (Kim et al., 2017). While heterologous expression and genome editing have elucidated SPS functions in model species, their metabolic regulation in maize remains poorly understood.

While the functional significance of *SPS* genes in primary and secondary metabolism has been well established across plant species, research on maize *SPS* remains at a nascent stage. Current literature predominantly focuses on model organisms (Kim et al., 2017); systematic investigations of maize *SPS* genes remain conspicuously absent. Transgenic and mutant analyses revealed that lines overexpressing *ZmSPS2* exhibited elevated α-tocopherol content alongside reduced γ-tocopherol levels, resulting in increased α/γ-tocopherol ratios (Feng et al., 2025). These findings suggest that functional dissection of *ZmSPS2* could be helpful to illuminate regulatory mechanisms of maize tocopherol biosynthesis, thereby informing quality improvement strategies.

As an integral component of multi-omics approaches, metabolomics bridges genotype-phenotype relationships and provides novel insights for systems biology when integrated with genomics, transcriptomics, and proteomics data (Gong et al., 2023; Wu, Gao, Zhang, Liu, & Hu, 2024). In this study, the *Zmsps2* mutants of W22 were generated via a Mu transposon-mediated approach (Feng et al., 2025). Comparative metabolomic analysis of leaves and kernels from W22 wild-type and the *Zmsps2* mutant will reveal significant impacts of the *Zmsps2* mutation on metabolic networks.

## 2 Materials and methods

### 2.1 Materials

The W22 and *Zmsps2* mutant were cultivated in the Zengcheng Teaching and Research Experimental Base of South China Agricultural University in September 2024. Plants were arranged in alternating wide-narrow row spacing (wide rows: 0.8 m; narrow rows: 0.3 m), with double-row planting (10 plants per row). Standard agronomic practices were implemented throughout the growth cycle. Samples were collected at 20 DAP from ear leaves and kernels. Each biological replicate comprised pooled tissues from three individual plants (1 g per sample), with five independent replicates per genotype to minimize individual variation. Samples were flash-frozen in liquid nitrogen for 2 min and stored at −80 °C. Subsequently, the samples for LC-MS analysis were analyzed by Personal Biotechnology Co., Ltd. (Shanghai, China).

### 2.2 Widely targeted metabolomics analysis

Metabolite extraction and identification followed Chen et al. (2022). Briefly, freeze-dried leaf and kernel tissues were ground (30 Hz), and 50 mg of homogenized powder was extracted with 1200 μL of pre-chilled 70% methanol containing internal standards. After vortexing (six cycles) and centrifugation, supernatants were filtered for liquid chromatography and mass spectrometry (LC-MS) analysis using a Thermo Ultimate 3000 system equipped with an ACQUITY UPLC® HSS T3 (150 × 2.1 mm, 1.8 μm, Waters) column maintained at 40 °C.

Gradient elution of analytes was performed for positive ion mode: 0.1% formic acid in water (C) and 0.1% formic acid in acetonitrile (D); for negative ion mode: 5 mM ammonium formate in water (A) and acetonitrile (B), at a flow rate of 0.25 mL/min. A 2 μL injection of each sample was conducted following equilibration. The solvent B gradient was applied linearly as follows: from 0 to 1 min, 2% B/D; from 1 to 9 min, 2% to 50% B/D; from 9 to 12 min, 50% to 98% B/D; from 12 to 13.5 min, 98% B/D; from 13.5 to 14 min, 98% to 2% B/D; and from 14 to 20 min, 2% D for the positive ion model (14 to 17 min, 2% B for the negative ion model). The mass spectrometry conditions were as follows: ESI-MSn experiments were conducted on a Thermo Q Exactive Focus mass spectrometer, with a spray voltage of 3.5 kV in positive mode and −2.5 kV in negative mode. The sheath gas and auxiliary gas were set at 30 and 10 arbitrary units, respectively, with a capillary temperature of 325 °C. Full scans were performed on the Orbitrap analyzer over a mass range of *m*/*z* 81–1000 at a mass resolution of 70,000. Data-dependent acquisition (DDA) MS/MS experiments utilized HCD scanning, with a normalized collision energy of 30 eV. Dynamic exclusion was applied to eliminate superfluous information from the MS/MS spectra.

### 2.3 Metabolite identification and quantification

Data were processed using XCMS v3.12.0, with metabolite annotation based on retention time, mass accuracy (<10 ppm), MS/MS fragmentation patterns, and collision energy matching against in-house and public databases (Gu et al., 2018; Luo, Deng, Yuan, Deng, & Jin, 2017). Unit variance-scaled data were subjected to hierarchical clustering analysis using the R package ComplexHeatmap (v1.0.12). Partial least squares-discriminant analysis (PLS-DA) was performed using MetaboAnalystR (OPLSR.Anal function). Differential metabolites were identified based on variable importance in projection (VIP > 1) and statistical significance (*P* < 0.05). Pathway enrichment analysis was performed using clusterProfiler (v4.6.0) with Kyoto Encyclopedia of Genes and Genomes (KEGG) annotations. One-way ANOVA analysis was performed using SPSS V19.0 software, and the graphs were plotted using GraphPad Prism v8.0 software.

## 3 Results

### 3.1 Metabolite analysis in leaves and kernels of maize

**Fig. 1:**
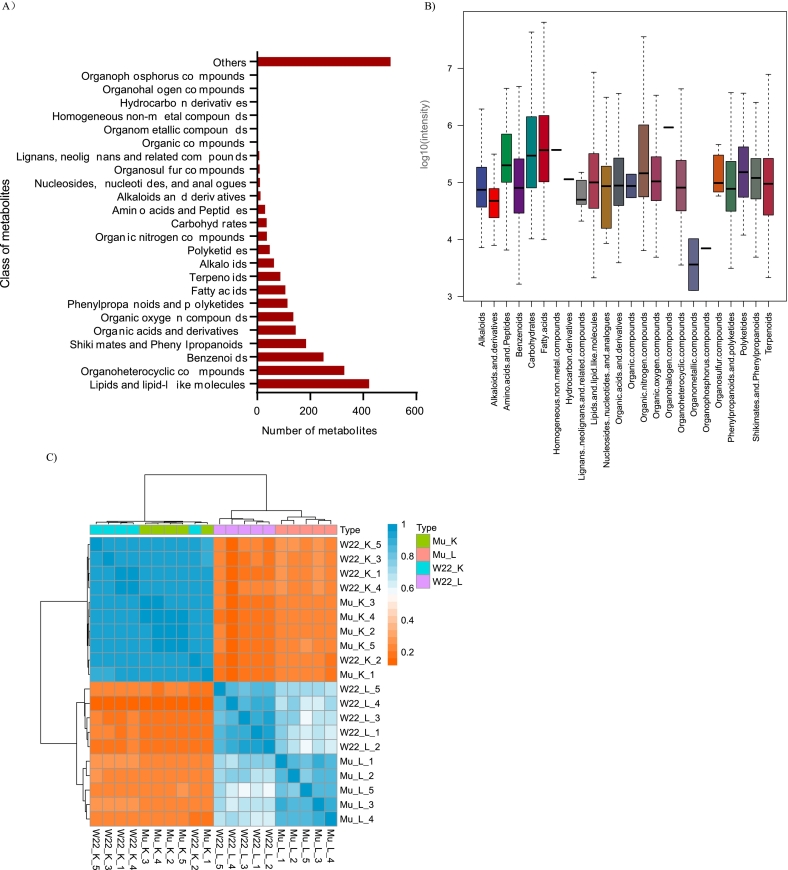
Metabolite classes and relative contents in the leaves and kernels of W22 and *Zmsps2* mutant. A: Categories of determined Metabolites in W22 and *Zmsps2* mutant. B: Range and distribution of relative peak values of metabolites (log_10_(intensity)) in W22 (*ZmSPS2*) and *Zmsps2* mutant. C: Correlation analysis of 20 samples of W22 and *Zmsps2* mutant.

For metabolomic profiling, leaf and kernel samples were collected from wild-type (W22) and *Zmsps2* mutant maize plants at 20 DAP (Fig. 1A). A total of 2531 metabolites were detected and characterized into 22 chemical classes (Table S1). The most abundant category was lipids and lipid-like molecules (249 metabolites, 16.67%), followed by organoheterocyclic compounds (219, 12.96%) and benzenoids (159, 9.88%). Other detected classes included organic acids and derivatives, phenylpropanoids, alkaloids, terpenoids, carbohydrates, and nucleotides, among others. The predominance of lipid-related compounds was consistent with previous metabolomic studies conducted in maize leaves (Li et al., 2024).

In terms of relative abundance across compound classes, carbohydrates and fatty acids exhibited the highest intensity, indicating they were the most abundant classes, while organometallic compounds showed the lowest intensity (Fig. 1B). The ten most abundant metabolites were γ-Linolenic acid, α-Linolenic acid, 1-Palmitoyl-2-hydroxy-sn-glycero-3-phospho-(1′-rac-glycerol), Dodecanedioic acid, Linoleic acid, 13(*S*)-HpOTrE, N-Cyclohexyl-N′-(1H-tetraazol-5-yl)urea, 3-[(1E,3E)-Hepta-1,3-dienyl] pentanedioic acid, 9-oxo-11-(3-pentyl-2-oxiranyl)-10E-undecenoic acid, and Oleamide (Fig. S1A and B). No significant differences were observed in the relative abundance of these 10 metabolites between the W22 and *Zmsps2* mutant. Correlation analysis revealed that metabolite profiles between kernels of the wild-type and mutant were highly correlated, while those between leaves also showed relatively low correlation. Heatmap analysis using differential metabolites revealed that a greater number of differential metabolites were identified in leaves (Fig. S2). Additionally, correlations among biological replicates were higher than those between the W22 and *Zmsps2* mutant groups (Fig. 1C).

### 3.2 Differential metabolite analysis in leaves

**Fig. 2:**
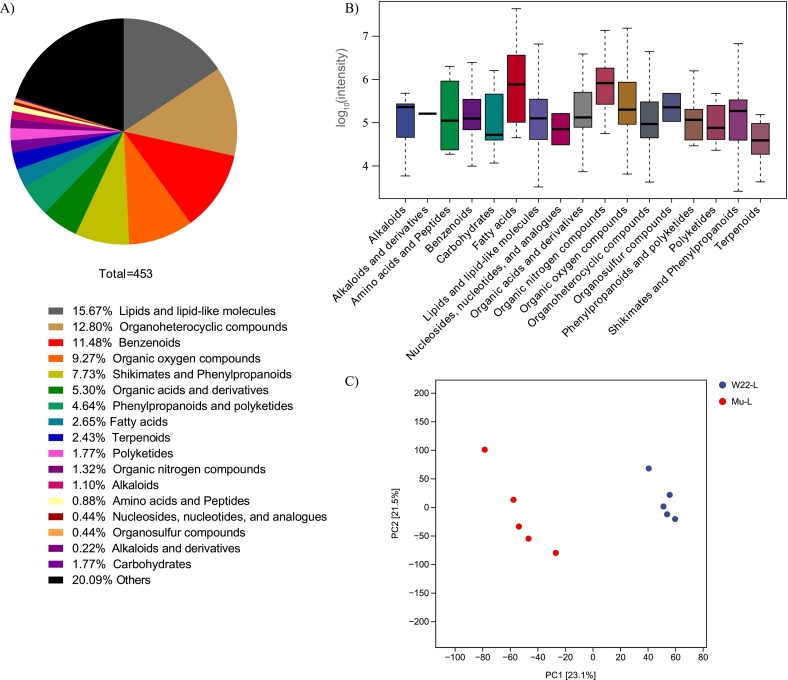
Distribution of identified differential metabolites and PLS-DA in wild-type and *Zmsps2* mutant leaves. (A) Category distribution of the 453 identified differential metabolites. (B) Range and distribution of differential metabolites in W22 and *Zmsps2* mutant lines. (C) PLS-DA of leaf metabolites from wild-type (red) and *Zmsps2* mutant (blue) plants. (For interpretation of the references to colour in this figure legend, the reader is referred to the web version of this article.)

A metabolomic analysis was performed on leaves of W22 and *Zmsps2* mutant at 20 DAP, and 453 differential metabolites were identified (Fig. 2A, Table S2). These metabolites were classified into 17 categories, including lipids and lipid-like molecules (71), organoheterocyclic compounds (58), benzenoids (52), organic oxygen compounds (42), shikimates and phenylpropanoids (35), organic acids and derivatives (24), phenylpropanoids and polyketides (21), fatty acids (12), terpenoids (11), carbohydrates (8), polyketides (8), organic nitrogen compounds (6), alkaloids (5), amino acids and peptides (4), nucleosides/nucleotides/analogues (2), organosulfur compounds (2), and alkaloids and derivatives (1) (Fig. 2B). An additional 91 metabolites remained unclassified. These metabolites predominantly participate in central metabolic pathways and certain secondary metabolic processes.

Among the 453 differential metabolites, the five most abundant in wild-type leaves were 9-oxo-11-(3-pentyl-2-oxiranyl)-10E-undecenoic acid, Oleamide, Methanone [4-hydroxy-1-[2-(4-morpholinyl)ethyl]-1H-indol-3-yl]-1-naphthalenyl-, Dinorprostaglandin E1, and 3-(benzoyloxy)-2-hydroxypropyl β-D-glucopyranosiduronic acid. In *Zmsps2* mutant leaves, the top five most abundant metabolites were 9-oxo-11-(3-pentyl-2-oxiranyl)-10E-undecenoic acid, Oleamide, 4-Hydroxy-3-oxo-1,3-dihydronaphtho[2,3-*c*]furan-5-yl β-D-glucopyranoside, Dodecanoic acid, and Dinorprostaglandin E1. These lists comprised seven unique metabolites, which were categorized into two groups: those highly abundant in the *Zmsps2* mutant [Dodecanoic acid (M72) and 4-hydroxy-3-oxo-1,3-dihydronaphtho [2,3-c] furan-5-yl β-D-glucopyranoside (M1035)] and those more abundant in W22 (Fig. S3). PLS-DA based on the relative abundance of differential metabolites revealed a clear separation between the metabolomic profiles of W22 and *Zmsps2* mutant leaves. The first two components explained 23.1% and 21.5% of the total variance, respectively (Fig. 2C).

### 3.3 Differential metabolite analysis in kernels

**Fig. 3:**
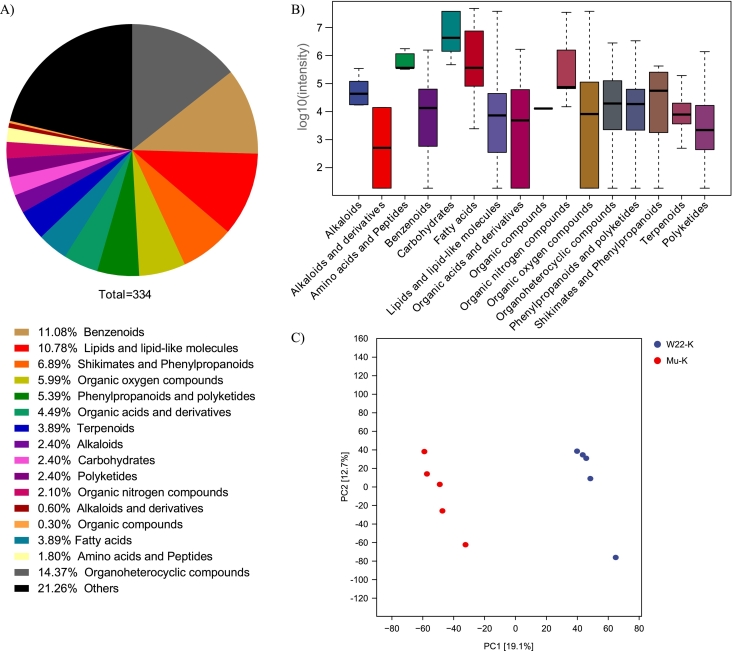
Distribution, relative abundance, and PLS-DA of identified differential metabolites in wild-type and *Zmsps2* mutant kernels. (A) Category distribution of the 334 identified differential metabolites. (B) Range and distribution of differential metabolites between W22 and *Zmsps2* mutant in kernels. (C) PLS-DA of kernel metabolites from wild-type (red) and mutant (blue) plants. (For interpretation of the references to colour in this figure legend, the reader is referred to the web version of this article.)

Metabolomic profiling was performed on kernels at 20 DAP, and 334 differential metabolites were identified between W22 and *Zmsps2* mutants (Fig. 3A, Table S3). These metabolites were categorized into 16 classes, including organoheterocyclic compounds (48), benzenoids (37), lipids and lipid-like molecules (36), shikimates and phenylpropanoids (23), organic oxygen compounds (20), phenylpropanoids and polyketides (18), organic acids and derivatives (15), fatty acids (13), terpenoids (13), alkaloids (8), carbohydrates (8), polyketides (8), organic nitrogen compounds (7), amino acids and peptides (6), alkaloids and derivatives (2), and organic compounds (1). An additional 71 metabolites remained unclassified. These metabolites are primarily involved in central metabolic pathways and certain secondary metabolic processes, with fatty acids and carbohydrates showing the highest relative abundance. These lists comprised six unique metabolites, which were categorized into two groups: highly abundant in the *Zmsps2* mutant [M132 (Linoleic acid)] and those more abundant in wild type (Fig. S4). Among the 334 differential metabolites, the five most abundant in wild-type kernels were linoleic acid, trehalose, palatinose (hydrate), melibiose, and sucrose (Fig. 3B). In kernels of the *Zmsps2* mutants, the most abundant metabolites were melibiose, sucrose, turanose, trehalose, and palatinose (hydrate).

PLS-DA based on the relative abundance of differential metabolites revealed distinct metabolomic profiles between W22 and *Zmsps2* mutants in kernels. The first two components accounted for 19.1% and 12.7% of the total variance, respectively (Fig. 3C).

### 3.4 Enrichment analysis of differential metabolites in leaves

**Fig. 4:**
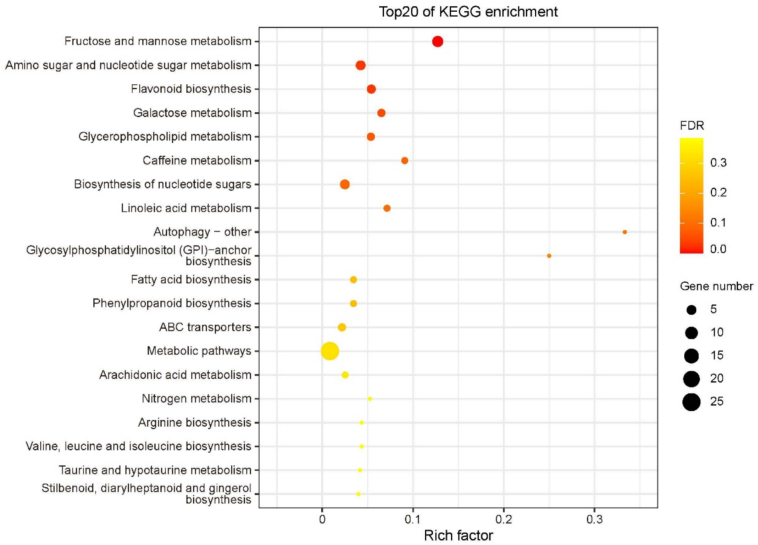
KEGG enrichment analysis of differentially accumulated metabolites in leaves.

Enrichment analysis of the 453 differential metabolites identified in leaves revealed four significantly enriched metabolic pathways (Fig. 4): zma00051 (Fructose and mannose metabolism), zma00520 (Amino sugar and nucleotide sugar metabolism), zma00941 (Flavonoid biosynthesis), and zma00052 (Galactose metabolism). The zma00051 pathway contained seven differential metabolites: fructose 2-phosphate, fructose 1-phosphate, mannose 6-phosphate, sorbose, glucose, mannose 1-phosphate, and allose. The first three metabolites were up-regulated, while the latter four were down-regulated. In the zma00520 pathway, five differential metabolites were identified: mannose 6-phosphate, glucose, mannose 1-phosphate, galactose, and fructose, with the first three up-regulated and the last two down-regulated. The zma00941 pathway included four differentially accumulated metabolites—catechin, epicatechin, isoliquiritigenin, and chlorogenic acid—all of which were down-regulated. Similarly, three metabolites in the zma00052 pathway showed down-regulated expression.

### 3.5 Enrichment analysis of differential metabolites in kernels

**Fig. 5:**
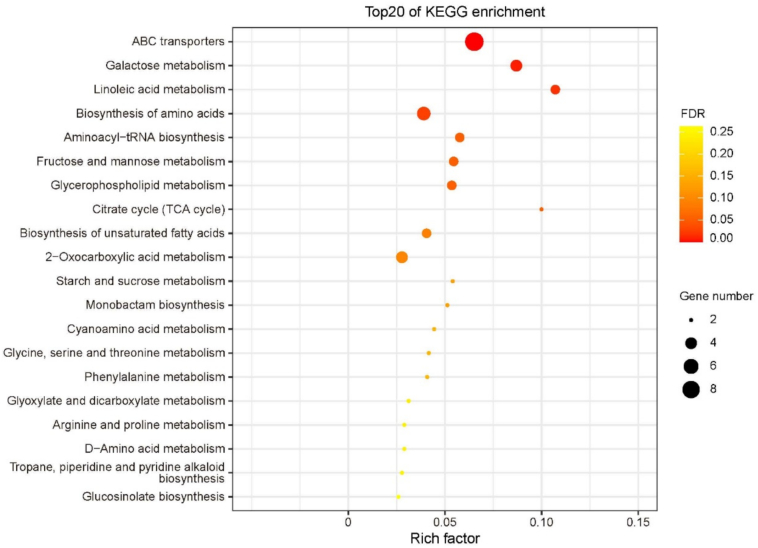
KEGG enrichment analysis of differential metabolites between W22 and *Zmsps2* mutant in kernels at 20 DAP.

**Fig. 6:**
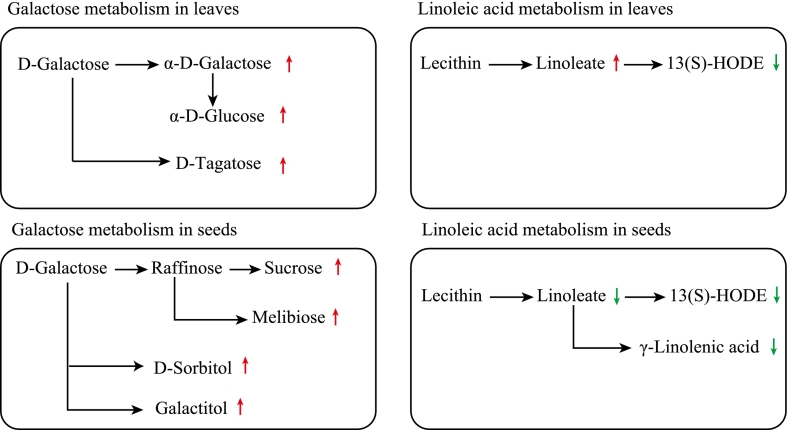
Altered galactose and linoleic acid metabolic pathways in leaves and kernels of wild-type and *Zmsps2* mutant plants, respectively. Metabolites with increased abundance are marked with red arrows, while those with decreased abundance are indicated by green arrows. (For interpretation of the references to colour in this figure legend, the reader is referred to the web version of this article.)

Four significantly enriched metabolic pathways were identified based on the 334 differential metabolites between W22 and *Zmsps2* mutant, including zma02010 (ABC transporters), zma00052 (Galactose metabolism), zma00591 (Linoleic acid metabolism), and zma01230 (Biosynthesis of amino acids) (Fig. 5). The zma02010 pathway contained nine differential metabolites: melibiose, sucrose, trehalose, sn-glycerol 3-phosphate, valine, betaine, arginine, mannitol, and glucitol. Among these, the first eight metabolites were up-regulated, while glucitol was down-regulated. The zma00591 pathway included three metabolites associated with linoleic acid metabolism: 13(*S*)-HODE, linoleic acid, and γ-linolenic acid, which showed decreased accumulation. Finally, five metabolites in the zma01230 pathway—valine, isocitric acid, citric acid, arginine, and tyrosine—were consistently up-regulated. A commonly significantly enriched pathway (zma00052) was also identified in kernels, and four metabolites (melibiose, sucrose, glucitol, and galactitol) were up-regulated. (Fig. 6).)

### 3.6 Metabolic pathway alterations induced by Zmsps2 mutation

Enrichment analysis revealed that the galactose metabolism pathway (zma00052) was commonly and significantly enriched in both leaves and kernels of *Zmsps2* mutants. In leaves, mutation of the *ZmSPS2* gene activated galactose metabolism, leading to an increase in galactose, which was rapidly converted to α-D-galactose and further accumulated as α-d-glucose and D-tagatose. In kernels, the *ZmSPS2* mutation redirected galactose metabolism toward storage and protection. D-galactose was efficiently converted into raffinose, promoting the accumulation of sucrose, melibiose, D-sorbitol, and galactitol within kernels (Fig. 6).

The commonly non-significantly enriched metabolic pathway associated with the *ZmSPS2* mutation was linoleic acid metabolism (zma00591). Linoleic acid metabolism exhibited dynamic changes: enhanced breakdown of phosphatidylcholine generated more linoleic acid, but its downstream oxidized product, 13(*S*)-HODE, was significantly reduced in leaves. In contrast, linoleic acid metabolism was broadly suppressed—from phosphatidylcholine to linoleic acid, γ-linolenic acid, and 13(*S*)-HODE—all showing declining trends (Fig. 6). This may reflect a shift in lipid metabolic emphasis toward triglyceride storage during seed maturation, which may reflect a shift in lipid metabolic focus toward triglyceride storage during kernel maturation, thereby avoiding lipid peroxidation.

## 4 Discussion

### 4.1 Impact of Zmsps2 on maize leaves

Metabolomic analysis of maize leaves revealed that mutation of *ZmSPS2* significantly reshaped the metabolic profile of leaves at 20 DAP. Among the 453 differential metabolites, lipids and lipid-like molecules (71 compounds) and organoheterocyclic compounds (58 compounds) constituted the most abundant categories, followed by benzenoids (52 compounds) and organic oxygen compounds (42 compounds). These changes indicate that the mutation primarily affected membrane lipid metabolism, the synthesis of aromatic compounds, and redox-related pathways. Notably, the differential accumulation of secondary metabolites such as phenylpropanoids/polyketides (35 compounds), terpenoids (11 compounds), and alkaloids (5 compounds) suggests that the mutation may influence plant stress resistance by modulating the synthesis of defensive metabolites (Li et al., 2025; Murphy et al., 2021).

Functional analysis of key differential metabolites revealed potential mechanisms of physiological regulation. The persistently high accumulation of 9-oxo-11-(3-pentyl-2-oxiranyl)-10E-undecenoic acid (an oxylipin derivative) and oleamide (a fatty acid amide) in both wild-type and mutant plants suggests their possible involvement in fundamental stress response pathways. Metabolites that specifically accumulated in the mutant included 4-hydroxy-3-oxo-1,3-dihydronaphtho[2,3-*c*]furan-5-yl glucoside and dodecanoic acid. The former may be associated with oxidative stress protection, while the latter likely reflects a shift in energy storage forms.

The PLS-DA model confirmed significant metabolomic alterations in the mutant. These changes are consistent with the extensive connectivity within the maize metabolic networks (Li et al., 2025): lipid remodeling may affect chloroplast membrane stability; shifts in phenylpropanoids indicate activation of the phenylalanine ammonia-lyase pathway; and fluctuations in terpenoids and alkaloids suggest a rebalancing of defense metabolism (Kim et al., 2021; Murphy et al., 2021). The presence of 91 unclassified metabolites further implies that the mutation of *ZmSPS2* may modulate metabolic pathways through currently undefined mechanisms.

### 4.2 Impact of Zmsps2 on maize kernels

Differences in high-abundance metabolites between wild-type and mutant plants revealed potential metabolic nodes affected by the mutation of *ZmSPS2*. The consistent accumulation of mannose suggests a possible role in maintaining osmotic balance or buffering carbon supply, analogous to the regulation of starch synthesis by *ZmTPS9* in maize—both of which are directly linked to reserve accumulation in kernels (Hu et al., 2021). Meanwhile, the reduction in fatty acid content, such as linoleic acid, may reflect an imbalance in energy metabolism, highlighting the sensitivity of fatty acid metabolism to the reallocation of energy flux. These metabolic changes support a central role for *ZmSPS2* in coordinating carbohydrate and lipid metabolism. The PLS-DA results further highlighted alterations in the kernel metabolome induced by the mutation of *ZmSPS2*. Such systemic modifications align with observations from evolutionary studies in maize (Xu et al., 2019b).

### 4.3 Impact of Zmsps2 on the metabolic pathways

The metabolic changes triggered by mutation of *ZmSPS2* displayed notable organ-specific patterns. In leaves, alterations were predominantly enriched in sugar metabolism-related pathways (zma00051, zma00520, zma00052) and secondary metabolite synthesis routes (zma00941), with bidirectional regulation of phosphorylated sugar intermediates being particularly prominent. For instance, within the fructose and mannose metabolism pathway (zma00051) pathway, the upregulation of phosphorylated monosaccharides such as fructose 2-phosphate contrasted sharply with the downregulation of free monosaccharides like allose, suggesting that the mutant may maintain sugar homeostasis by enhancing phosphorylation levels (Giesbrecht, Bonn, & Furtauer, 2025). This regulatory pattern, combined with the synchronous downregulation of four key metabolites in the flavonoid biosynthesis pathway (zma00941), reflects that photosynthetic products are preferentially directed toward fundamental sugar metabolism at the expense of secondary metabolite synthesis (Girija et al., 2022). Notably, galactose metabolism (zma00052) was enriched in both leaves and kernels, yet the direction of metabolite changes was completely opposite, underscoring the determining role of the organ-specific microenvironment in modulating metabolic networks (Farcuh et al., 2017).

In contrast to leaves, the core alterations in mutant kernels were concentrated in transmembrane transport (zma02010) and enhanced anabolic metabolism (zma01230). The significant upregulation of eight sugar and amino acid metabolites in the ABC transporters pathway, along with accumulation of sugar alcohols such as galactitol in zma00052, indicates activation of the transmembrane solute transport system (Chen et al., 2025; Gu et al., 2025). Cross-organ comparative analysis revealed that differential metabolites in leaves and kernels were co-enriched in the zma00052 pathway. This shared enrichment suggests that mutation of *ZmSPS2* may regulate source–sink plasticity by altering sugar metabolism.

The regulation of galactose metabolism and linoleic acid metabolism by the *ZmSPS2* mutation exhibited tissue specificity. In leaves, the mutation likely maintains carbon metabolic homeostasis by adjusting the soluble sugar pool. In kernels, galactose flux was primarily directed toward the raffinose pathway, promoting the accumulation of sucrose, melibiose, and protective sugar alcohols. This metabolic shift contributes to seed storage stability. Although linoleic acid metabolism did not reach significant enrichment levels, its dynamic changes revealed organ-specific adaptations. In leaves, enhanced phosphatidylcholine decomposition increased linoleic acid production, yet the significant reduction in its downstream oxidized product, 13(*S*)-HODE, may indicate suppression of oxidative metabolic pathways. This selective regulation could be associated with leaf adaptive mechanisms against oxidative stress. In kernels, the entire metabolic cascade from phosphatidylcholine to γ-linolenic acid and 13(*S*)-HODE was suppressed. This comprehensive downregulation may reflect a resource reallocation toward triacylglycerol storage during late kernel development in *ZmSPS2* mutants, simultaneously reducing the peroxidation risk of polyunsaturated fatty acids to safeguard seed viability (Dong et al., 2025). These results underscore the key role of *ZmSPS2* in coordinating lipid allocation across different organs.

Metabolomic enrichment analysis of W22 and *Zmsps2* mutant kernels revealed significant downregulation of zma00591, and upregulation of zma01230, zma00052 and zma02010, suggesting a multifaceted impact on the tocopherol biosynthetic precursors. Previous studies have reported that loss of *ZmSPS2* function increases γ-tocopherol accumulation and reduces the α/γ-tocopherol ratio (Feng et al., 2025); however, the association between these metabolic alterations and tocopherol biosynthesis has not been reported. Further investigation is needed to clarify how *ZmSPS2* regulates tocopherol biosynthesis.

## 5 Conclusions

This study reveals the extensive impact of *ZmSPS2* mutation on metabolic networks in maize leaves and kernels at 20 DAP. A total of 2531 metabolites were identified, with 453 and 334 differential metabolites detected in leaves and kernels, respectively. These alterations were significantly enriched in pathways such as galactose metabolism, amino sugar metabolism, and linoleic acid metabolism. The mutation induced tissue-specific metabolic changes: in leaves, it activated galactose metabolism, promoting the accumulation of galactose and its derivatives while reducing levels of the oxidized metabolite 13(*S*)-HODE; in kernels, it redirected metabolism toward the synthesis and accumulation of protective sugars such as raffinose and melibiose, as well as sugar alcohols. By modulating carbon allocation, the mutation of *ZmSPS2* enhances energy supply in leaves while improving accumulation of protective sugars in kernels. This study provides new insights into the regulation of metabolic profiles by mutation of *ZmSPS2*.

## CRediT authorship contribution statement

**Jianting Lin:** Writing – original draft, Data curation. **Yanchao Du:** Writing – review & editing, Formal analysis, Data curation. **Haoxuan Jiang:** Validation, Investigation. **Huating Zhao:** Validation, Investigation. **Bo Wang:** Writing – review & editing, Conceptualization. **Faqiang Feng:** Funding acquisition, Conceptualization.

## Declaration of competing interest

The authors declare that they have no known competing financial interests or personal relationships that could have appeared to influence the work reported in this paper.

## Data Availability

Data will be made available on request.
