# Supplementary material for: Comparative metabolomic analysis of leaves and kernels in wild type and Zmsps2 mutant
Source: Food Chem (Oxf). 2025 Dec 11;12:100343. doi: 10.1016/j.fochms.2025.100343 (PMC12769848; doi:10.1016/j.fochms.2025.100343)
Supplement: Supplementary Fig. S1-4 [file mmc1.docx]

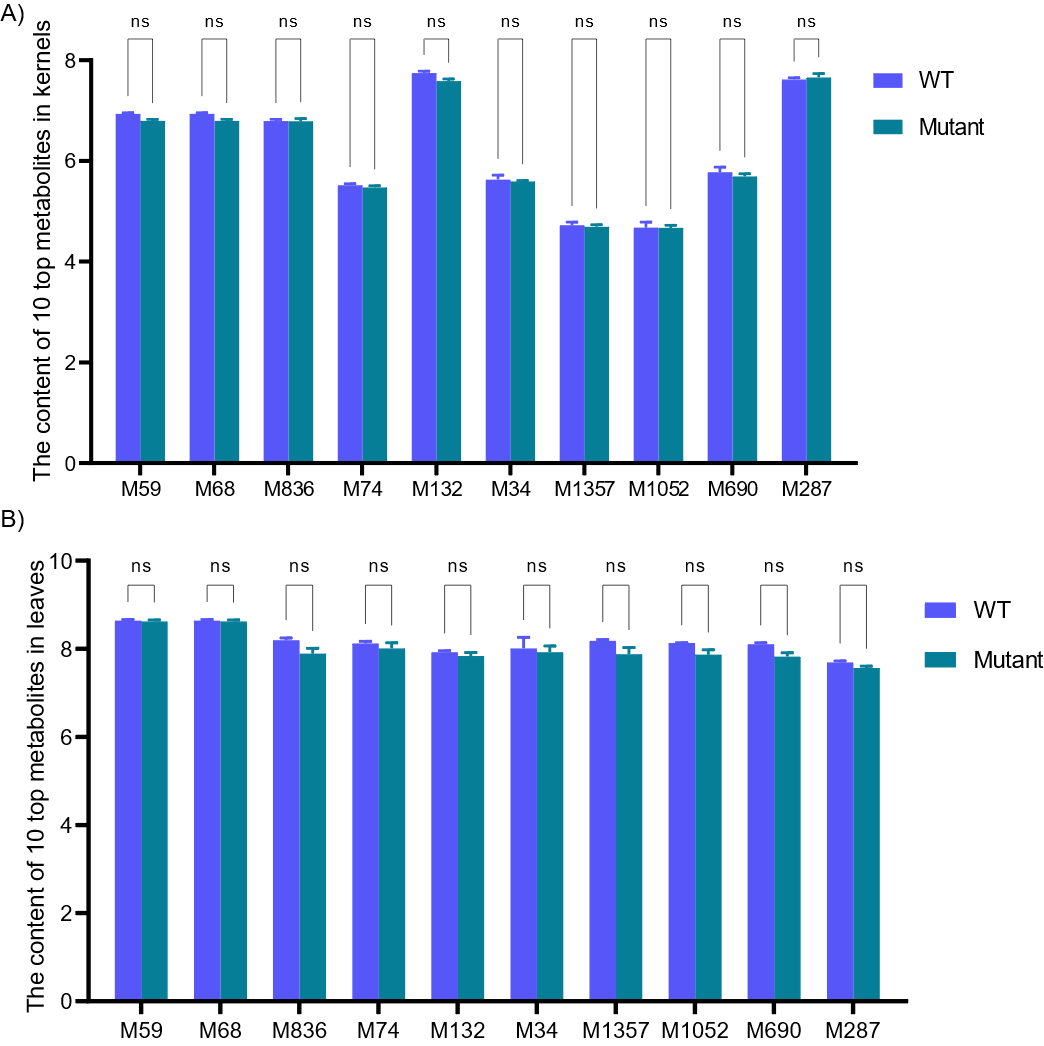


Fig. S1. The relative content of 10 top metabolites in kernels (A) and leaves (B). M59, gamma-Linolenic acid; M68, alpha-Linolenic acid; M836, 1-Palmitoyl-2-hydroxy-sn-glycero-3-phospho-(1'-rac-glycerol); M74, Dodecanedioic acid; M132, Linoleic acid; M34, 13(S)-HpOTrE; M1357, N-Cyclohexyl-N'-(1H-tetraazol-5-yl)urea; M1052, 3-[(1E,3E)-Hepta-1,3-dienyl]pentanedioic acid; M690, 9-Oxo-11-(3-pentyl-2-oxiranyl)-10E-undecenoic acid; M287, Oleamide. The relative content of metabolites was transformed using log_10_(intensity).


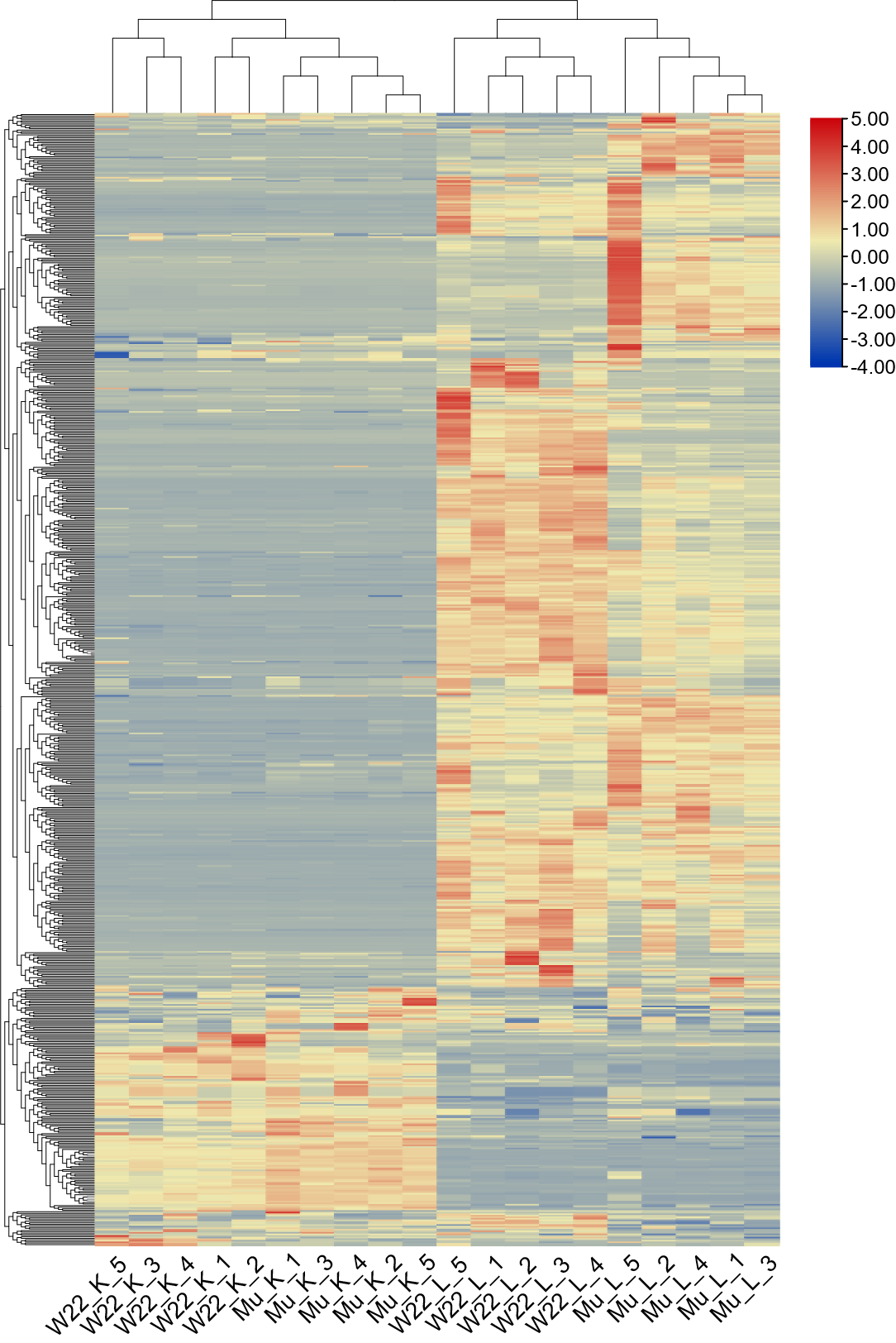


Fig. S2 The heatmap of differential metabolites (DMs) in both leaves and kernels. The threshold of VIP >1 and *P* <0.05 was used to screen DMs between W22 and the mutant at 20 days after pollination. The relative content data were standardized by rows.


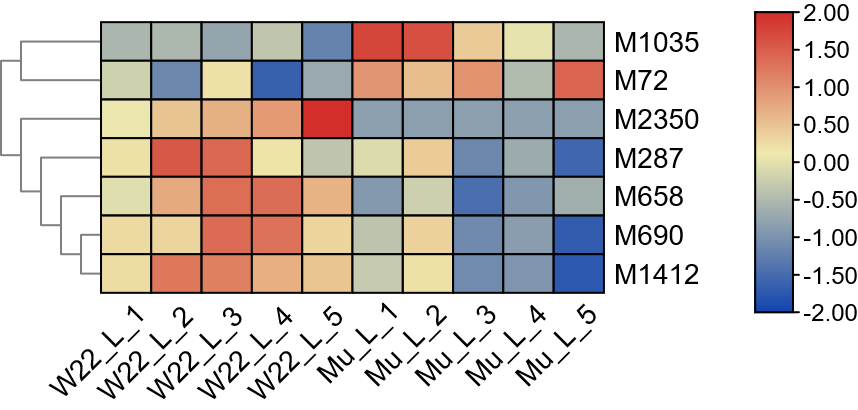


Fig. S3 The heatmap of 7 shared DMs between the W22 and *Zmsps2* mutant in leaves. The threshold of VIP >1 and *P* <0.05 was used to screen DMs between W22 and the mutant at 20 days after pollination. The relative content data were standardized by rows. M1035, 4-Hydroxy-3-oxo-1,3-dihydronaphtho[2,3-c]furan-5-yl β-D-glucopyranoside; M72, Dodecanoic acid; M2350, Methanone [4-hydroxy-1-[2-(4-morpholinyl)ethyl]-1H-indol-3-yl]-1-naphthalenyl-; M287, Oleamide; M658, 3-(Benzoyloxy)-2-hydroxypropyl β-D-glucopyranosiduronic acid; M690, 9-Oxo-11-(3-pentyl-2-oxiranyl)-10E-undecenoic acid; M1412, Dinorprostaglandin E1.


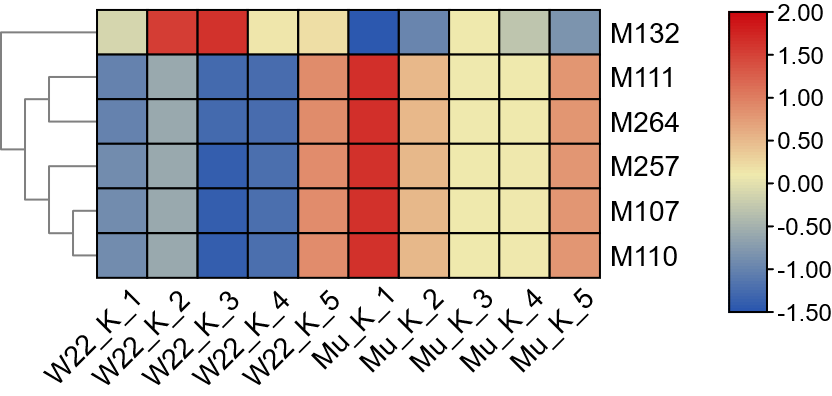


Fig. S4 The heatmap of 6 shared DMs between the W22 and *Zmsps2* mutant in kernels. The threshold of VIP >1 and *P* <0.05 was used to screen DMs between W22 and the mutant at 20 days after pollination. The relative content data were standardized by rows. M132, Linoleic acid; M111, Trehalose; M264, Palatinose (hydrate);M257, Turanose; M107, Melibiose; M110, Sucrose.
